# COVID-19 Mediated by Basigin Can Affect Male and Female Fertility

**DOI:** 10.22074/ijfs.2020.134702

**Published:** 2020-10-12

**Authors:** Soodeh Mahdian, Maryam Shahhoseini, Ashraf Moini

**Affiliations:** 1. Department of Cellular and Molecular Biology, Faculty of Biological Sciences, North Tehran Branch, Islamic Azad University, Tehran, Iran; 2. Reproductive Epidemiology Research Center, Royan Institute for Reproductive Biomedicine, ACECR, Tehran, Iran; 3. Department of Genetics, Reproductive Biomedicine Research Center, Royan Institute for Reproductive Biomedicine, ACECR, Tehran, Iran; 4. Department of Cell and Molecular Biology, School of Biology, College of Science, University of Tehran, Iran; 5. Department of Endocrinology and Female Infertility, Reproductive Biomedicine Research Center, Royan Institute for Reproductive Biomedicine, ACECR, Tehran, Iran; 6. Breast Disease Research Center (BDRC), Tehran University of Medical Sciences, Tehran, Iran; 7. Department of Gynecology and Obstetrics, Arash Women’s Hospital, Tehran University of Medical Sciences, Tehran, Iran

**Keywords:** Basigin, CD147, COVID-19, Fertility

## Abstract

Coronavirus disease 2019 (COVID-19) prevalence has caused many problems in society and disrupted many regular aspects of life. COVID-19 contains major structural proteins that among them, S protein can promote fusion of the viral and cellular membranes and facilitate the entry of coronavirus into the host cells. Basigin (BSG) is one of the most important receptors for COVID-19 that mediates its entry to host cells. Also, Basi- gin has an important role in male and female reproduction. Basigin is expressed in the uterus and plays an important role during embryo implantation and needed for successful implantation. Therefore, disruption or inhibition of Basigin causes to a weakness in embryo implantation. Therefore, if a woman or a man is infected with COVID-19, it is recommended that they do not attempt to conception until their treatment is complete. It is also recommended tests for COVID-19 be performed on infertile couples before using assisted reproductive technology (ART).

Basigin (BSG), also known as CD147 is a glycosylated transmembrane protein in human cells. Basigin is a potent inducer for matrix metalloproteinases and vascular endothelial growth factor. Also Basigin is an important regulator of cell metabolism (1).

## Role of Basigin in male and female reproduction

BSG is expressed in Sertoli cells, Leydig cells and germ cells and is recognized as a critical factor for spermatogenesis. So, disruption or inhibition of Basigin causes to spermatogenesis failure (2). Expression of BSG has been confirmed in the stroma, cumulus and granulosa cells of ovary. BSG mRNA and protein were detected in granulosa cells in follicles at all stages of development and also in the corpora lutea (1). Some data suggest that BSG may play a role during the follicle development and corpus luteum formation (3).

## Basigin and embryo implantation

Basigin is expressed in the uterus and plays an important role during embryo implantation, in the way that embryonic expression of BSG is needed for successful implantation (4, 5). Therefore, disruption or inhibition of Basigin causes to a weakness in embryo implantation of embryos.

## Basigin and COVID-19 invasion

BSG is a cellular receptor of COVID-19 which can mediate the entry of virus into the host cells (6). It also has an impact on certain infectious diseases such as malaria, Neisseria meningitides, and HIV-1 (7). COVID-19 contains the major structural proteins Spike (S), envelope (E), membrane (M), and nucleocapsid (N), among them, S protein can promote the fusion of viral and cellular membranes (8). So, it facilitates the entry of coronavirus into the host cells. It is reported that COVID-19 creates a novel invasion route with binding of S protein to BSG through which it invades host cells. This novel invasion route for COVID-19 provides a new target for anti-viral drug development (9).

Finally, it is concluded that Basigin is necessary for normal fertility in both males and females. Concerning the invasion route of COVID-19 mediated by Basigin, it is hypothesized that the COVID-19 infection can potentially effect on reproduction. Therefore, if a woman or a man is infected with COVID-19, it is recommended that they do not attempt to conception until their treatment is complete. It is also recommended tests for COVID-19 be performed on infertile couples before using assisted reproductive technology (ART).
